# Development of a multiplex ddPCR assay for simultaneous absolute quantification of bacterial, fungal, and human DNA

**DOI:** 10.1371/journal.pone.0341560

**Published:** 2026-02-20

**Authors:** Guohong Huang, Torrey L. Gallagher, Gregory J. Tsongalis, Joel A. Lefferts

**Affiliations:** Department of Pathology and Laboratory Medicine, Dartmouth Hitchcock Medical Center, Lebanon, New Hampshire, United States of America; Children's National Hospital, George Washington University, UNITED STATES OF AMERICA

## Abstract

Molecular methods in clinical and research applications frequently encounter complex mixtures of human and microbial DNA, sometimes alongside environmental or reagent contaminants. In metagenomic studies, the presence of host contamination poses a significant challenge, reducing the assay sensitivity of microbial detection and characterization. Different host-depletion and microbial enrichment platforms have been developed to reduce or eliminate host DNA in samples predominantly composed of human DNA. To establish an effective method to assess the efficacy of host DNA depletion or microbial enrichment platforms, this study aimed to develop a multiplex, broad-range 16S/18S ribosomal DNA droplet digital PCR (rDNA ddPCR) assay capable of simultaneously quantifying bacterial and fungal DNA, along with a human housekeeping gene, RPP30 (Ribonuclease P/MRP Subunit P30). Genomic DNA from key representatives of Gram-positive bacteria, Gram-negative bacteria, and fungi was tested in broad-range 16S/18S rDNA duplex (16S/RPP30 or 18S/RPP30) and triplex (16S/18S/RPP30) ddPCR assays to determine optimal assay conditions, specificity, and sensitivity. This assay demonstrated high sensitivity, specificity, and reproducibility, with detection limits of approximately 3 copies/µL for the 16S target (0.5 pg *Staphylococcus aureus* gDNA) and 1–2 copies/µL for the 18S target (16 fg of *Candida albicans* gDNA) in both duplex and triplex formats. Within a defined range, a linear relationship was observed between microbial DNA input and 16S/18S rDNA copy number by ddPCR. Furthermore, different commercial ddPCR master mixes had contrasting effects on the amplitudes of positive 16S/18S droplet clusters. As a proof of concept for the assay’s utility in metagenomic studies, we demonstrated that one extraction kit achieved more efficient depletion of human DNA and better enrichment of microbial DNA. In summary, we developed a multiplex, broad-range 16S/18S ddPCR assay with high sensitivity and specificity, which holds promise as a QA/QC (Quality Assurance/Quality Control) platform in metagenomic studies and other research settings.

## Introduction

When employing molecular methods for clinical or research applications, samples frequently contain variable mixtures of human and microbial DNA, sometimes supplemented by environmental or reagent contaminants. Better understanding the types and amounts of DNA present during testing can lead to higher quality molecular studies.

Metagenomic sequencing, a powerful platform for microbiome studies in both biomedical and environmental contexts [1–11], offers a broad ranged approach to investigate microbial communities and their functions more comprehensively than targeted sequencing. It facilitates novel species identification and the detection of other genetic markers related to phenotype [12,13]. One major focus of metagenomic studies is the investigation of the human microbiome in health and disease [1,2,5,14–23]. The presence of excessive host DNA poses a significant challenge in many human specimens, such as nasal, oral, skin swabs, whole blood, and biopsy tissues [3,24]. Alternatively, sequencing of specimens with very low amounts of microbial DNA can result in an over-representation of human DNA. While the presence of excessive host DNA may be tolerated in targeted sequencing platforms, it can overwhelm pathogen signals in metagenomics studies which sequence all DNA in the sample, leading to reduced sensitivity or skewed results in microorganism detection, particularly in studies involving low microbial biomass [2,25–29].

To address this, various methods have been developed to selectively deplete host DNA and/or enrich microbial DNA before or after DNA extraction, using physical or combined physical-chemical approaches [30]. Despite certain limitations, host depletion and microbial enrichment methods have proven valuable in characterizing microbial profiles in samples predominantly composed of human DNA [3,20,24,30–34]. As a result, these techniques are increasingly adopted in clinical metagenomics. However, an effective method to assess the efficacy of host DNA depletion or microbial enrichment platforms is still lacking. Most studies rely on final sequencing results to evaluate the performance of extraction kits, which is both costly and time-consuming. While some studies use separate qPCR assays to quantify bacterial and human DNA, these assays typically provide relative, rather than absolute, quantification, and the presence of fungal species is often overlooked. Since fungi can cause infections, either independently or as part of “mixed infections” [1,18,22,35,36], it is vital to monitor fungal DNA levels along with bacterial DNA in metagenomic studies.

Since its commercialization in 2011, ddPCR has been increasingly adopted for rigorous DNA quality control in different settings, particularly in applications requiring high sensitivity and absolute quantification. Unlike traditional quantitative PCR (qPCR), which relies on a standard curve and relative quantification, ddPCR provides a digital, absolute count of target nucleic acid molecules. Numerous studies have demonstrated equivalent or superior performance of ddPCR over qPCR [37–51].

The high precision and absolute quantification capability of ddPCR make it an ideal tool for assessing critical quality attributes of a DNA sample. For instance, ddPCR has been successfully used for assessing the quantity and quality of degraded DNA samples [52,53], quality assurance for infectious disease molecular testing [54], quality control of minimal residue disease [55] and DNA methylation analyses [56], water quality monitoring and pathogen surveillance [50,57–59], and quality control of raw health food materials [60].

In this study, we developed a multiplex, broad-range ddPCR assay capable of simultaneously quantifying bacterial 16S rDNA, fungal 18S rDNA, and the human housekeeping gene RPP30 (Ribonuclease P/ MRP Subunit P30) in a single reaction. In metagenomic applications, the assay enables absolute quantification of both microbial and human DNA, providing critical insights into sample composition and quality, which is essential for evaluating the effectiveness of host depletion or microbial enrichment platforms. Additionally, as the microbial DNA-to-human DNA ratio is a key factor in successful microbial identification [3,28], this assay can provide insight and guidance for subsequent steps. To the best of our knowledge, this is the first study to describe a simultaneous, universal approach for quantifying bacterial and fungal DNA alongside a human housekeeping gene in a single reaction, making it a valuable platform for monitoring human DNA contamination and providing guidance for protocol optimization in metagenomic studies.

## Materials and methods

### Genomic DNA

Genomic DNA from key representatives of Gram-positive bacteria, Gram-negative bacteria, and fungi was purchased from ATCC (American Type Culture Collection) and reconstituted in low EDTA TE buffer. These pathogens included common Gram-positive bacterium *Staphylococcus aureus* (ATCC # BAA-1556D-5), common Gram-negative bacterium *Pseudomonas aeruginosa* (ATCC # 9027D-5), and common opportunistic fungus *Candida albicans* (ATCC # 14053D-5). Another common Gram-negative bacterial species, *Escherichia coli*, was obtained as a strain mix from a collaborator’s lab at Dartmouth-Hitchcock Medical Center and the bacterial DNA was extracted from a liquid culture with the DNeasy Blood & Tissue kit (QIAGEN), following the manufacturer’s protocol tailored for gram-negative bacteria. Human genomic DNA derived from fresh frozen autopsy lung tissue was isolated using the DNeasy Blood & Tissue kit, following the manufacturer’s spin-column protocol for tissue samples.

Total DNA concentration was determined using the Qubit dsDNA High-sensitivity Quantitation Assay Kit (Thermo Fisher Scientific) on the Qubit 4.0 fluorometer.

### ddPCR assay primers and probes

The primers and probes targeting the 16S region (bacteria) or 18S region (fungi) of rDNA were based on conserved domains in all bacterial or fungal species, according to previous publications [61,62]. These primers and probes were synthesized by IDT (Integrated DNA Technologies) with the 16S and 18S probes specifically labeled with FAM for detection purposes. For the detection of human DNA (hDNA), the RPP30 gene was targeted with commercial primer/probe mix including a HEX-labeled probe (Bio-Rad Cat # 10031243). Detailed information regarding the primer and probe sequences can be found in S1 Table.

### Microbial DNA detection by ddPCR

The presence of bacterial 16S or fungal 18S DNA was detected separately or simultaneously using a QX200 Droplet Digital PCR system (Bio-Rad), according to protocols in publications [61,62] and the manufacturer’s manual. Unless specified, the ddPCR reaction consisted of a 22 µL reaction volume with ddPCR Supermix for probes (Bio-Rad, Cat # 1863023, named Master mix A in this study), primers/probes for 16S and/or 18S (IDT, customized), primers/probes for RPP30 (Bio-Rad) (see below for ranges of primer/probe concentrations), 0.5 mM betaine (Sigma-Aldrich), 1.0 mM EDTA (Sigma-Aldrich), 140 IU/µL CviQI restriction enzyme (New England Biolabs), 4 µL of DNA sample, and molecular grade nuclease-free water.

We initially conducted experiments to determine the optimal conditions for primer/probe concentrations and the annealing/extension temperatures for both duplex (16S/RPP30 or 18S/RPP30) and triplex (16S/18S/RPP30) assays. The concentrations of microbial primers tested spanned from 225 to 900 nM, while those of the probes ranged from 62.5 to 250 nM. Optimal detection of both bacterial and fungal DNA was achieved with a combination of 16S primers/probes at 900/250 nM and 18S primers/probes at 450/125 nM. Consequently, this combination was chosen for subsequent ddPCR assays due to its robust performance. Temperature gradient experiments were conducted within the range of 50 to 65^o^C.

To enhance the distinction between positive microbial clusters and negative droplets, we also tested the impact of the ddPCR Multiplex Supermix (Bio-Rad, Cat # 12005909, named Master mix B in this study) and 300 mM dithiothreitol (DTT, Bio-Rad).

Twenty microliters (20 µL) of each ddPCR reaction mixture underwent droplet generation utilizing a Bio-Rad Automated Droplet Generator, followed by PCR in a C1000 Touch™ Deep-Well Thermal Cycler (Bio-Rad) with the following cycling parameters unless specified otherwise: 95^o^C for 5 min, 40 cycles (with ramp rate 2^o^C s^-1^) of 94^o^C for 30 s and 56^o^C for 1 min, a final step of 98°C for 10 minutes, and cooled to 4°C for at least 30 minutes before being analyzed on the Bio-Rad QX200 droplet reader using the QuantaSoft v1.7.4 software. The ddPCR results were expressed as copies/µL in the 20 µL ddPCR reaction.

### Analytic specificity and sensitivity

The analytical specificity of the primers and probes was first evaluated *in silico* using the NCBI BLASTn program to exclude cross-reactivity of the primer/probes to off-target sequences. Subsequently, the specificity of each assay was empirically tested against off-target DNA samples. To establish the Limit of Blank (LoB) for each target, negative samples (comprised of water and off-target DNA samples) were utilized, following the methodology outlined in a previous publication [63].

To obtain the detection limit of bacterial 16S rDNA, *S. aureus*, one of the most common bacterial pathogens, was used. Bacterial DNA was diluted in nuclease-free water from 1 pg/µL to 1 fg/µL following a 2-fold dilution series and 4 µL of each dilution was tested by ddPCR as described above, both in the duplex format of 16S/RPP30 and in the triplex format of 16S/18S/RPP30. Each sample was tested in triplicate. The final dilution at which all replicates of pathogen DNA could be reliably distinguished from the negative controls was determined as the detection limit of the assay [64]. Similarly, the detection limit for 18S was determined using *C. albicans* DNA, with a modification that the dilutions were conducted in water and 10 ng/µL herring sperm DNA (Promega) in parallel due to the instability of *C. albicans* DNA in water at very low concentrations (< 1 pg/µL).

### Comparison of two commercial DNA extraction kits using synovial fluid samples

For comparison of microbial DNA extraction efficiency from two commercial host-depletion kits, we used archived synovial fluid samples from consented total joint arthroplasty patients collected under Dartmouth-Hitchcock Institutional Review Board protocol STUDY02000173, from 12/03/2019–06/23/2023. During data analysis, the authors had access to potentially identifiable information, including patient names and medical records. However, all data were anonymized prior to analysis, and no identifiable information was retained in the final dataset.

Upon collection, synovial fluid samples from consented total joint arthroplasty patients were diluted with equal volume of PBS and stored at -80^o^C until DNA extraction. DNA was extracted with Zymo HostZERO Microbial DNA Kit (Cat # D4310) or Molzym MolYsis Complete 5 kit (Cat # D-321–050) kits following the manufacturers’ instructions. Each extraction started with the same volume of synovial fluid, with an elution volume of 20 µL for the Zymo kit and 100 µL for the Molzym kit. The extracted DNA was quantified by Qubit dsDNA High-sensitivity Quantitation Assay Kit as described above. For each DNA sample extracted, 1 µL was tested using the triplex ddPCR assay.

### Statistical analysis

The ddPCR results underwent statistical analysis using the paired t-test, with a p-value of <0.05 deemed statistically significant. Additionally, the linearity of the data was assessed using the R^2^ value in Excel, serving as a measure of the data’s linear fit.

## Results and discussion

### Annealing/extension temperature optimization

**Fig 1:**
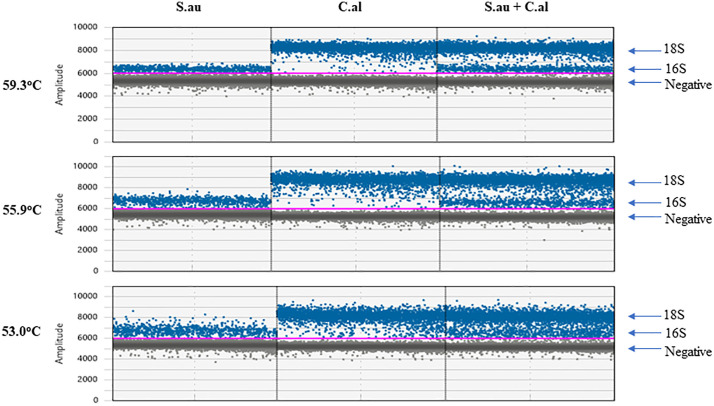
The impact of annealing/extension temperature on 16S and 18S detection in ddPCR. S.au, *S. aureus.* C.al, *C. albicans*. S.au and C.al gDNA were diluted in water and 4 pg of each sample was tested individually or combined. The 1D amplitude plots under different temperatures were shown. Blue dots represent positive droplets, gray dots represent negative droplets, and pink lines represent thresholds.

In the temperature gradient experiment for the triplex ddPCR assay, amplification efficiency declined significantly above 62°C, while temperatures below 53°C resulted in fewer positive droplets (S1A Fig). Importantly, the RPP30 assay showed no cross-reactivity with non-human DNA (S1B Fig). As illustrated in Fig 1, within the 53–59°C range, increasing the temperature improved cluster tightness, with 55.9°C yielding the best separation between positive and negative droplets for both the 16S and 18S targets. Notably, 18S-positive droplets consistently displayed higher fluorescence amplitudes than 16S, and microbial concentrations remained consistent across this temperature range. Based on these findings, 56°C was chosen as the annealing/extension temperature for all subsequent experiments.

### Specificity

**Fig 2:**
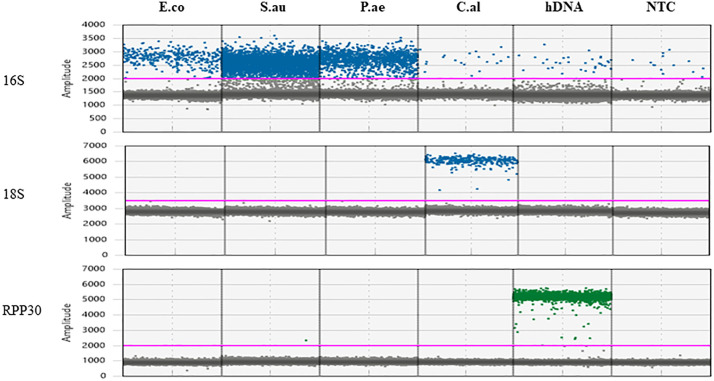
The specificity of the established microbial ddPCR assay. E.co, *E coli.* S.au, *S. aureus.* P.ae, *P. aeruginosa.* C.al, *C. albicans.* hDNA, human DNA isolated from fresh frozen lung tissue. NTC, No Template Control (water). Microbial DNA was diluted in water and 16 pg was tested for each microorganism. hDNA was tested at 10 ng. Note the background 16S level in C.al, hDNA and NTC was due to the presence of trace amount of *E. coli* DNA attributable to microbial DNA carry-over in the Taq polymerase.

The specificity of the assay was evaluated with off-target DNA samples. The 16S assay exhibited the capability to specifically detect 16S DNA in all bacterial species tested, but not in the fungal or human DNA; Similarly, the 18S and RPP30 assay was able to specifically detect fungal and human DNA, respectively, but not the bacterial DNA (Fig 2). Low-level detection of the 16S-positive droplets (approximately 1–2 copies/µl) was observed in all reactions not expected to contain the 16S target DNA sequence (fungal DNA, human DNA and NTC). This occurrence suggested potential cross-reactivity or low-level contamination, with an average Limit of Blank (LoB) around ~2 copies/µL across multiple runs.

This background amplification phenomenon is not uncommon and has been reported in other studies as well [64,65]. Upon consulting Bio-Rad technical support, we learned that the Supermix for probes contains trace amount of *E. coli* DNA as a byproduct of the manufacturing process. To mitigate this, background amplification can be subtracted from the ddPCR output to normalize the data. Alternatively, reagents specifically designed to be free of *E. coli* or other contaminating DNA—such as the ddPCR™ Supermix for Residual DNA Quantification (Bio-Rad, Catalog # 1864037), which was not used in this study—may be employed to minimize or eliminate background amplification.

### Sensitivity of the 16S assay

**Fig 3:**
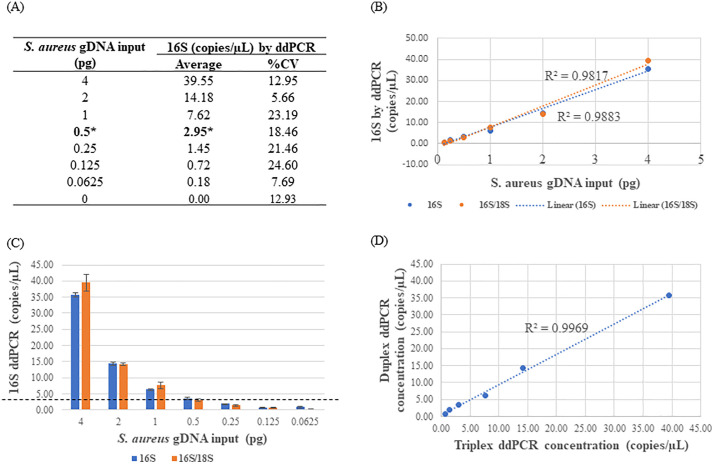
Detection of *S. aureus* 16S rDNA in duplex or triplex ddPCR. 2X serial dilutions of *S. aureus* gDNA were conducted in water and 4 µL of each dilution was assayed in triplicate. The data shown indicated average values. **(A)** The correlation between DNA input and the 16S concentration in triplex ddPCR. The data was normalized by background subtraction and the number in bold with * indicated LoD (Limit of Detection). %CV represents %(Coefficient of Variation). **(B)** Quantification linearity of 16S in duplex and triplex ddPCR. **(C)** Limit of detection estimation of *S. aureus* in duplex and triplex ddPCR. Error bars indicated standard deviation. Black line indicated LoD. **(D)** Correlation of 16S concentration in duplex and triplex ddPCR.

The sensitivity of the 16S assay was examined rigorously using *S. aureus* in both 16S and 16S/18S formats to determine whether the presence of off-target primer/probes impacted bacterial DNA detection by ddPCR. Comparable bacterial concentrations were observed in both duplex and triplex reactions, exhibiting a strong linear correlation with an R² of 0.997 (Fig 3D). Within DNA input ranging from 62.5 fg to 4 pg, a robust linear correlation was observed between DNA input and the 16S concentration by ddPCR (R^2^ ~ 0.98, Fig 3B). The assay’s detection limit was estimated at approximately 3 copies/µL, equivalent to 0.5 pg of DNA or roughly 10 bacterial cells as depicted in Fig 3 and S2 Fig. Notably, 0.5 pg DNA corresponds to about 66 genome equivalents in a 22 µL ddPCR reaction volume, considering that the *S. aureus* genomic DNA used contains 6 copies of the 16S rDNA gene per genome. Additionally, the 16S assay showed strong inter- and intra-assay reproducibility, as shown in S2 Table. Together, these results highlight the reliability and robustness of the 16S assay for sensitive microbial DNA detection via ddPCR.

### Sensitivity of the 18S assay

**Fig 4:**
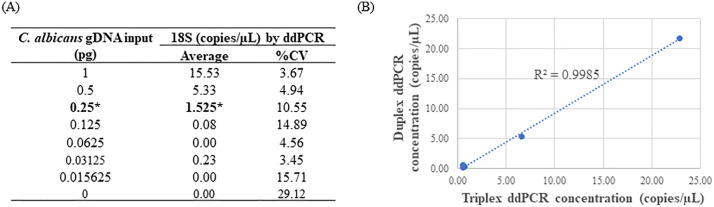
Detection of *C. albicans* 18S rDNA in duplex or triplex ddPCR. 2X serial dilutions of *C. albicans* gDNA were conducted in water and 4 µL of each dilution was assayed in triplicate. The data shown indicated average values. **(A)** The correlation between DNA input and the 18S concentration in triplex ddPCR. The numbers in bold with * indicated LoD values. DNA instability was observed as poor linearity was observed and higher 18S concentration was expected for each dilution. **(B)** Correlation of 18S concentration in duplex and triplex ddPCR.

**Fig 5:**
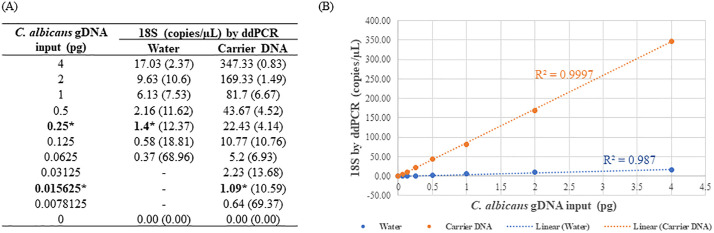
Effect of carrier DNA on fungal 18S detection by ddPCR. 2X series dilutions of *C. albicans* DNA were conducted in water or carrier DNA in parallel and assayed by duplex ddPCR in triplicate. **(A)** The correlation between DNA input and the 18S concentration by ddPCR. The data was displayed as average (%CV) and the numbers in bold with * indicated LoD values. **(B)** Quantification linearity of 18S by ddPCR in the presence or absence of carrier DNA.

The sensitivity assessment of the 18S assay involved testing with *C. albicans* DNA in both the 18S and 16S/18S formats. Similar to the observations in the 16S assay, the detected fungal concentrations were almost identical in the duplex and triplex assays with a linear correlation R^2^ value of 0.999 (Fig 4). Surprisingly, larger variations in 18S rDNA quantifications were noted in both duplex and triplex reactions compared to the 16S rDNA target across multiple independent experiments (S2 Table), indicating a potential stability issue of *C. albicans* DNA at extremely low concentrations (below 1 pg/µL). Therefore, an investigation was conducted to explore whether the presence of herring sperm DNA (utilized at 10 ng/µL) could stabilize *C. albicans* DNA. As depicted in Fig 5 and S3 Fig, in the presence of carrier DNA, significantly higher 18S concentrations were observed compared to equivalent dilutions in water (p < 0.001). Consequently, the 18S assay successfully detected *C. albicans* DNA at an input as low as 16 fg in the presence of carrier DNA and 250 fg without carrier DNA. Under both conditions, the estimated detection limit remained consistent at 1–2 copies/µL and a strong linear correlation existed between the fungal DNA input and the 18S rDNA concentration by ddPCR.

Validation of the ddPCR assay in both duplex (16S/RPP30 or 18S/RPP30) and triplex format (16S/18S/RPP30) allows flexibility for detecting bacterial or fungal species individually or simultaneously. It is important to note that the 16S copy number in bacterial genomes is typically much lower (only several copies per genome) compared to the 18S copy number in fungal genomes (up to hundreds or thousands of copies per genome), and both copy numbers can vary under different conditions [61,66–69]. Despite using lower 18S primers/probe concentrations and accounting for the larger genome of *C. albicans* versus *S. aureus*, the 18S assay remained more sensitive, particularly when *C. albicans* DNA was stabilized with carrier DNA. DNA instability in highly diluted samples is well recognized, and strategies such as maintaining high DNA concentration or adding carrier DNA have been shown to slow DNA degradation [70,71]. However, the differing stability of *C. albicans* and *S. aureus* DNA under low concentrations remain unclear. As demonstrated in this study, inclusion of carrier DNA could be considered to optimize assay sensitivity under such conditions.

### Master mix comparison

Despite the high sensitivity and specificity conferred by the ddPCR Supermix for Probes (Bio-Rad Cat # 1863023, Master mix A) in microbial DNA detection, the positive 16S droplets clustered closely to the negative droplets, suggesting a potential low efficiency of the 16S ddPCR reaction under the aforementioned conditions. To achieve better separation between positive and negative clusters, we explored the efficacy of another Bio-Rad optimized multiplex ddPCR mastermix, Multiplex Supermix (Bio-Rad Cat # 12005909, Master mix B), and the inclusion of 300mM DTT, an additive known for its enzyme-stabilizing properties in PCR when used at low concentration [72]. Both the Multiplex Supermix and DTT present an opportunity to enhance the performance of the ddPCR assay.

**Fig 6:**
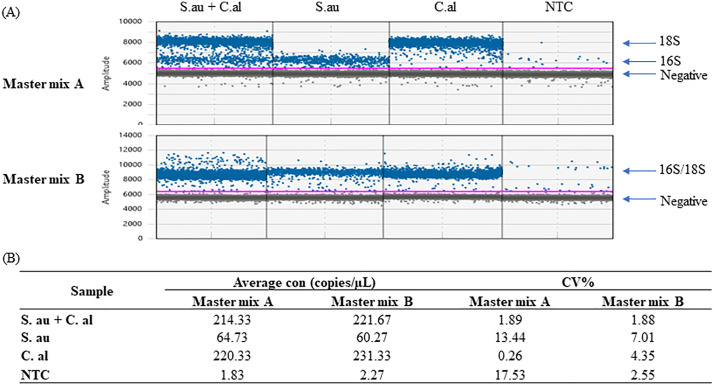
The impact of different master mixes on 16S and 18S detection by triplex ddPCR. Master mix A, ddPCR Supermix for Probes (No dUTP). Master mix B, ddPCR Multiplex Supermix. S.au, *S. aureus.* C.al, *C. albicans.* S.au and C.al gDNA was diluted in water and 4 pg of each sample was tested individually or combined, using Master mix A or **B.** The run was done in triplicate. **(A)** The 1D amplification amplitude plots. **(B)** The average microbial concentration and % CV of each sample.

**Fig 7:**
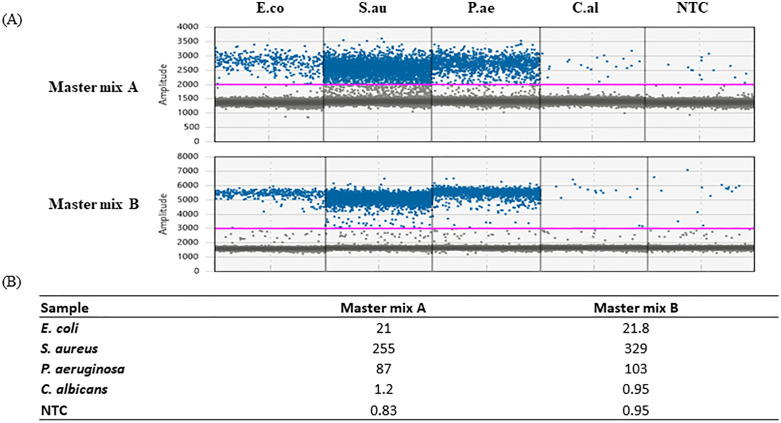
The impact of different master mixes on 16S ddPCR assay performance. E.co, *E. coli.* S.au, *S. aureus.* P.ae, *P. aeruginosa.* C.al, *C. albicans.* NTC, No Template Control (water). Microbial genomic DNA was diluted in water and 16 pg was tested for each microorganism. **(A)** The 1D amplification amplitude with Master mix A or B. **(B)** The comparison of 16S ddPCR concentration (copies/µL) with the two different Master mixes.

Notably, the introduction of the Master mix B augmented the amplitude of the 16S positive clusters but it concurrently decreased the amplitude of the 18S positive clusters. Consequently, instead of observing two distinct positive microbial populations with the Master mix A, only one positive population was discernible with Master mix B (**Fig 6**). Master mix B also appeared to maintain or even surpass the sensitivity of Master mix A, without compromising the assay’s overall specificity for microbial DNA (**Fig 7**). Further, Master mix B produced tighter and cleaner positive droplet clusters, coupled with a reduction in droplet noise between clusters across all three bacterial species tested. This improvement underscores the potential utility of Master mix B in refining ddPCR assay outcomes.

DTT stands as an optional component in multiplex ddPCR assays using the QX200 ddPCR system, with Bio-Rad recommending its addition to potentially optimize the performance of Master mix B. We conducted an experiment to evaluate the impact of DTT on the 16S/18S assay and found that the inclusion of 300mM DTT resulted in an increase in background amplitude and decreased the separation between positive and negative clusters (S4 Fig). Consequently, the utilization of DTT was not included in the final version of this 16S/18S ddPCR assay.

### Applications of the established assay in metagenomic studies – extraction kit comparison

As a proof of concept for the assay’s utility in metagenomic studies, we compared two DNA extraction kits for isolating microbial DNA from periprosthetic joint samples that had been confirmed to have bacterial infection by culture and/or sequencing methods. Extracted DNA was assessed by the established triplex ddPCR assay and our results revealed that Kit 2 consistently outperformed Kit 1 in depleting host (human) DNA and/or enriching microbial DNA, leading to higher 16S/RPP30 ratios in infected samples (Table 1). This highlights the utility of the 16S/18S ddPCR assay in evaluating the effectiveness of DNA extraction kits. Moreover, assuming a strong correlation between ddPCR results and metagenomic sequencing data, the multiplex ddPCR assay could serve as the foundation for a QA/QC platform. This platform, based on microbial DNA concentration and the microbial/human DNA ratio from the ddPCR assay, would provide guidance for subsequent steps and potentially eliminate the need for sequencing of non-infected samples, saving time and cost.

**Table 1:** Quantification of microbial and human DNA (copies/µL) in synovial fluid samples extracted by two different kits.

Sample Name	Kit 1			Kit 2		
	16S cp/µL	RPP30 cp/µL	16S/RPP30	16S cp/µL	RPP30 cp/µL	16S/RPP30
Sample A	–	1173	–	331	1574	0.21
Sample B	–	0.63	–	8.2	11.1	0.74
Sample C	–	14.7	–	11.8	32.9	0.36
Sample D	4.1	76.7	0.05	8	23.3	0.34
Sample E	124	450	0.28	793	140	5.66
Sample F	–	1130	–	7.9	39.3	0.20
Extraction control	–	–	–	–	–	–
NTC	–	–	–	–	–	–

Kit 1: Zymo HostZERO Microbial DNA Kit; Kit 2: Molzym MolYsis Complete 5 kit. Samples A – F were infected samples confirmed by metagenomic sequencing and/or culturing methods, while a dash (“-”) represented background microbial or RPP30 levels below the Limit of Detection (LoD). Since only bacterial species were present in the samples, the microbial DNA was labeled as 16S for simplicity.

### Other potential applications of the established assay

Beyond metagenomic applications, the ddPCR assay can be readily adapted as a contamination surveillance tool across diverse settings. Contamination from DNA extraction kits and other laboratory reagents is a well-documented issue that can significantly skew results if not properly identified and eliminated [25,26,28,73–81]. Therefore, to ensure sample integrity and accurate results, especially in studies involving low microbial biomass, careful experimental design and proper controls are essential [1,16,75,76,78,82,83]. As an example, in this study, unusually high bacterial concentrations were traced to contamination from a specific lot of the RPP30 primer/probe, demonstrating the assay’s value for reagent QC. Similarly, the assay can be used to monitor microbial contamination in human samples, environmental contexts, and medical devices. In one instance, when troubleshooting a failure of a clinical buccal epithelial sample submitted to our lab for germline genetic testing, the ddPCR assay revealed a predominance of bacterial and fungal DNA with significantly reduced human DNA in the sample, suggesting improper collection methods and storage conditions. Additionally, as highlighted in a recent review [50], ddPCR is gaining recognition as a valuable tool for water quality surveillance. This assay enables simultaneous detection of bacterial and fungal contaminants in drinking and waste water.

The established multiplex ddPCR assay can also serve as a QA/QC platform in certain clinical settings. In cases of microbial infections at sterile sites, such as heart valves and joints—particularly those involving non-culturable or slow-growing microbes—this assay could act as a frontline screening tool to check the presence or absence of bacterial and fungal species in suspected infections. It provides valuable complementary information to traditional culturing and sequencing methods. Since each platform has its own strengths and limitations, combining approaches can offer a more comprehensive understanding of the disease when necessary.

Furthermore, the DNA levels measured by the assay can also serve to normalize microbial or human DNA input and pipetting errors, which is essential for ensuring the accuracy and reproducibility of assays requiring equal sample input. Additionally, in diverse ecosystems where bacteria and fungi dominate, the assay can be used to assess the ratio of fungi to bacteria, offering valuable insights into the structure of the microbial community [46].

Overall, the examples described above highlight the multifaceted utility of simultaneously detecting bacterial, fungal, and human DNA, offering valuable insights into sample composition, DNA quality, and assay normalization in both research and clinical settings.

### Advantages and limitations of the ddPCR assay

The established multiplex ddPCR assay offers several advantages, including simplicity, speed, and cost-effectiveness. Results can typically be obtained within 3–4 hours, with an average per-sample cost of approximately $20–30, assuming access to ddPCR equipment (a droplet generator and reader) is available. In laboratories lacking ddPCR-specific equipment, the assay can still be validated within a qPCR framework, as the FAM/HEX probes used are compatible with standard qPCR platforms.

It is essential to acknowledge the limitations of the established multiplex ddPCR assay. One limitation is its broad-range detection approach, which does not allow for microbial identification at the genus or species level. Additionally, while the broad-spectrum primer/probes are designed to universally detect bacterial and fungal species, they may not be as conserved as expected [66,84] and new microbial species with 16S or 18S variants can evolve over time. Another potential drawback in some scenarios is the narrower linear dynamic range of ddPCR compared to quantitative PCR (qPCR), along with its inability to distinguish between viable and dead cells [64].

In conclusion, we have successfully developed a highly sensitive and specific multiplex ddPCR assay that enables the simultaneous, absolute detection of bacterial 16S, fungal 18S, and human RPP30 DNA. This assay holds great potential for a wide range of applications in both research and clinical settings, including but not limited to extraction kit comparison, assessment of sample quality and quantity in metagenomic studies, monitoring of contamination in samples, reagents, or environmental settings.

## Supporting information

S1 Table Primer/Probe information. (PDF)

S1 Fig Temperature gradient experiment on 16S and 18S detection by triplex ddPCR. 1: *S. aureus*; 2: *C. albicans*; 3: *S. aureus* and *C. albicans.* Microbial DNA was diluted in water, and 4 pg of each sample was tested individually or combined. 16S primer/probes were used at concentrations of 900/250 nM and those of the 18S were used at 450/125 nM.
(PDF)

S2 Fig 16S limit of detection (LoD) estimation in 16S or 16S/18S formats. Concentration plots of 16S under both formats were presented with the red lines indicating LoD. Serial dilutions of *S. aureus* gDNA were performed in water (2X dilution series), and 4 µL of selected dilutions were assayed in triplicate in parallel.
(PDF)

S2 Table Detection of *S. aureus* and *C. albicans* gDNA by ddPCR in multiple independent runs. (PDF)

S3 Fig Detection sensitivity of *C. albicans* in water (A) or carrier DNA (B). Series dilutions of *C. albicans* DNA were conducted in water or carrier DNA in parallel and assayed by duplex ddPCR in triplicate. The 1D amplitude plots were shown.
(PDF)

S4 Fig The impact of DTT on 16S and 18S detection in triplex ddPCR. S.au, *S. aureus.* C.al, *C. albicans.* S.au and C.al gDNA was diluted in water and 4 pg of each sample was tested individually or combined, in the presence or absence of DTT. The 1D (left) and 2D (right) amplitude plots were shown.
(PDF)

S1 Data Raw data. (XLSX)
