# Supplementary material for: Development of a multiplex ddPCR assay for simultaneous absolute quantification of bacterial, fungal, and human DNA
Source: PLoS One. 2026 Feb 20;21(2):e0341560. doi: 10.1371/journal.pone.0341560 (PMC12923063; doi:10.1371/journal.pone.0341560)
Supplement: S1 Table — (PDF) [file pone.0341560.s001.pdf]

S1 Table. Primer/Probe information

Target	Oligo ID	DNA sequence 5' to 3'	Amplicon Length (bp)	Supplier (Catalog Number)
16S rRNA	16S rRNA F*	AACAGGATTAGATACCCTGGTAG	200	Integrated DNA Technologies
	16S rRNA R*	GGTTCTKCGCGTTGCWTC		
	16S rRNA P*	6-FAM AACACTGCTCCACCGCT-MGBNFQ		
18S rRNA	18S rRNA F**	TTGGTGGAGTGATTTGTCTGCT	159	Integrated DNA Technologies
	18S rRNA R**	TCTAAGGGCATCACAGACCTG		
	18S rRNA P**	6-FAM TTAACCTACTAAATAGTGCTGCTAGC-BHQ1		
RPP30	ddPCR Copy Number Assay: RPP30, Human	MIQE Context: Hg19|chr10:92660373-92660495, 5' HEX, 3' Iowa Black FQ	67	Bio-Rad (10031243, Assay dHsaCP2500350)

MGBNFQ: Minor grove binder non fluorescent quencher; BHQ1: Black Hole Quencher-1; 6-FAM: 6-Carboxyfluorescein amidite; HEX: Hexachloro-fluoresein amidite
K=G/T, R=A/G, W=A/T (wobble bases); F=Forward, R=Reverse, P=Probe
* Adapted from Ziegler et al. 2019 (reference 21); ** Adapted from Li et al. 2019 (reference 39).
