# Supplementary figures and images for: Development of a multiplex ddPCR assay for simultaneous absolute quantification of bacterial, fungal, and human DNA
Source: PLoS One. 2026 Feb 20;21(2):e0341560. doi: 10.1371/journal.pone.0341560 (PMC12923063; doi:10.1371/journal.pone.0341560)

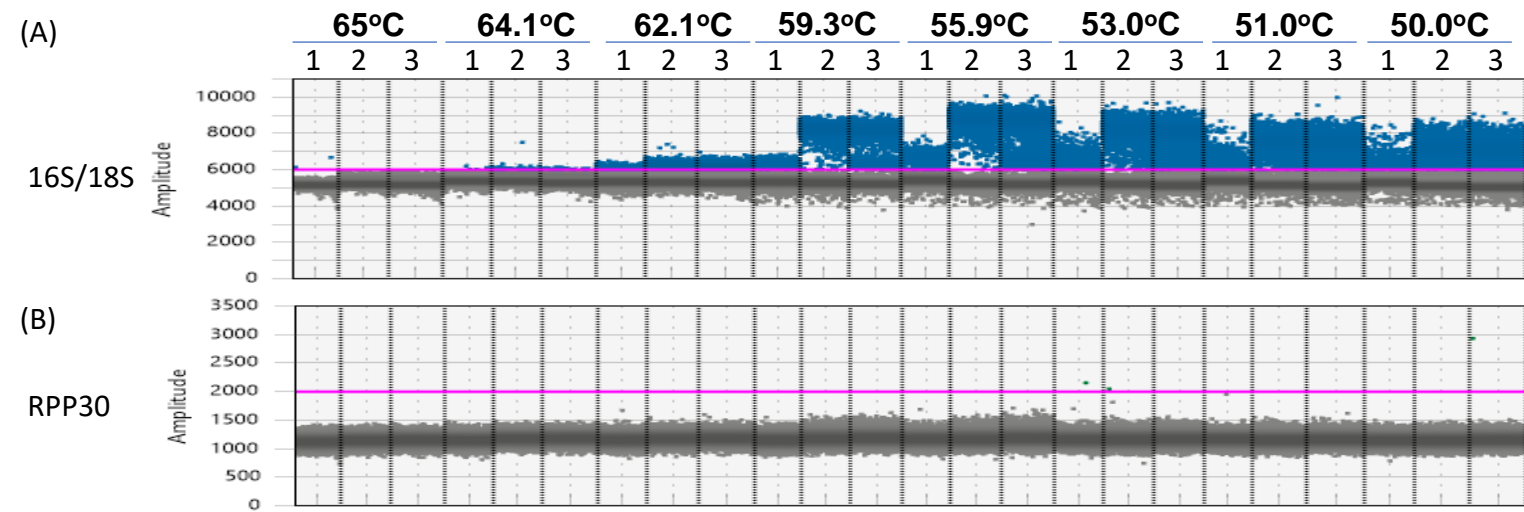

Supplement: S1 Fig — 1: S. aureus; 2: C. albicans; 3: S. aureus and C. albicans. Microbial DNA was diluted in water, and 4 pg of each sample was tested individually or combined. 16S primer/probes were used at concentrations of 900/250 nM and those of the 18S were used at 450/125 nM. (PDF) [file pone.0341560.s002.pdf]

16S format

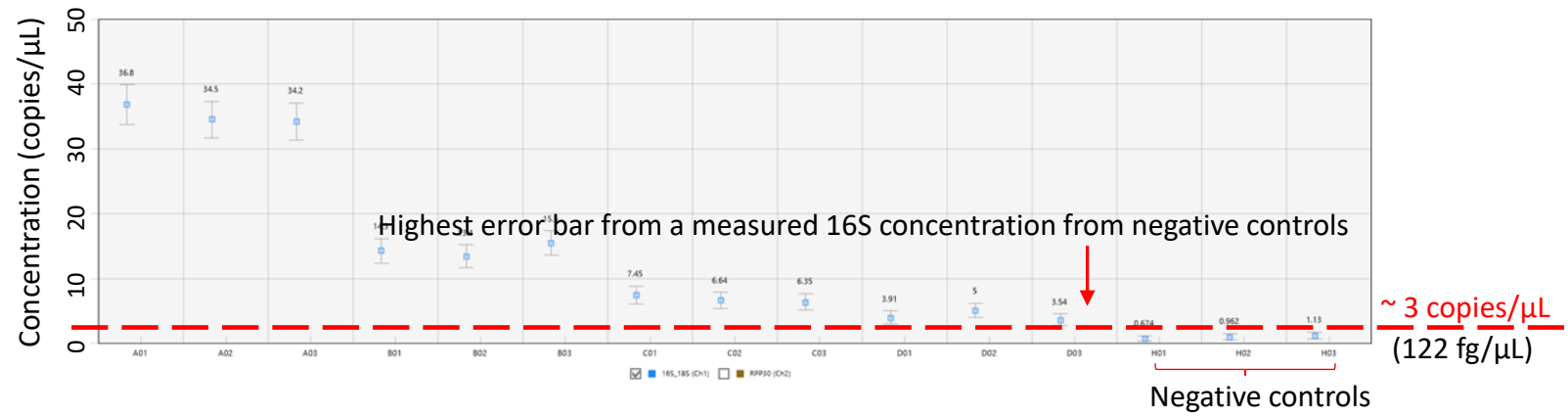

16S/18S format

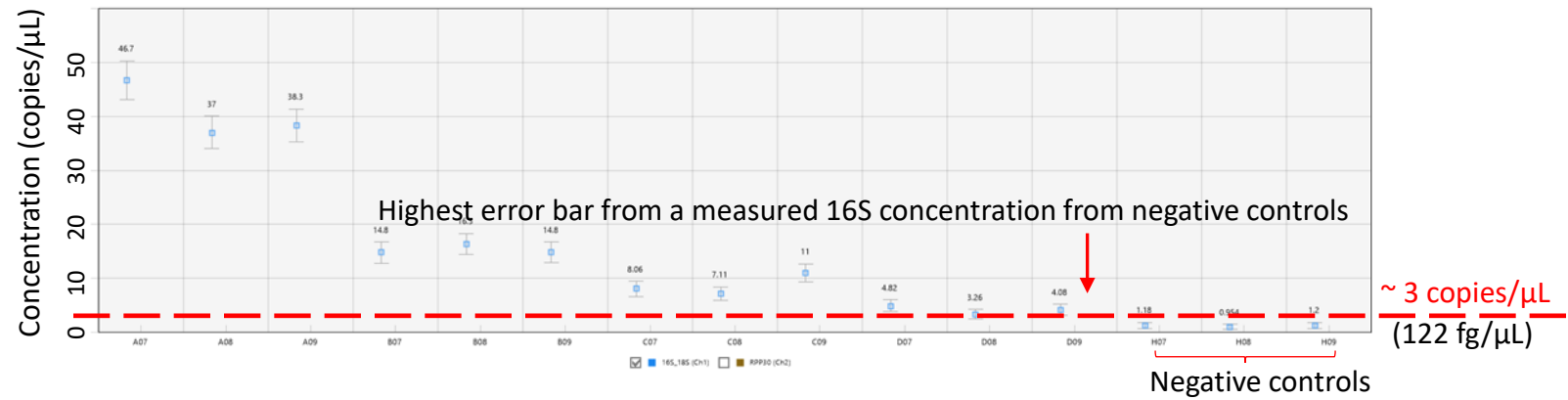

Supplement: S2 Fig — Concentration plots of 16S under both formats were presented with the red lines indicating LoD. Serial dilutions of S. aureus gDNA were performed in water (2X dilution series), and 4 µL of selected dilutions were assayed in triplicate in parallel. (PDF) [file pone.0341560.s003.pdf]

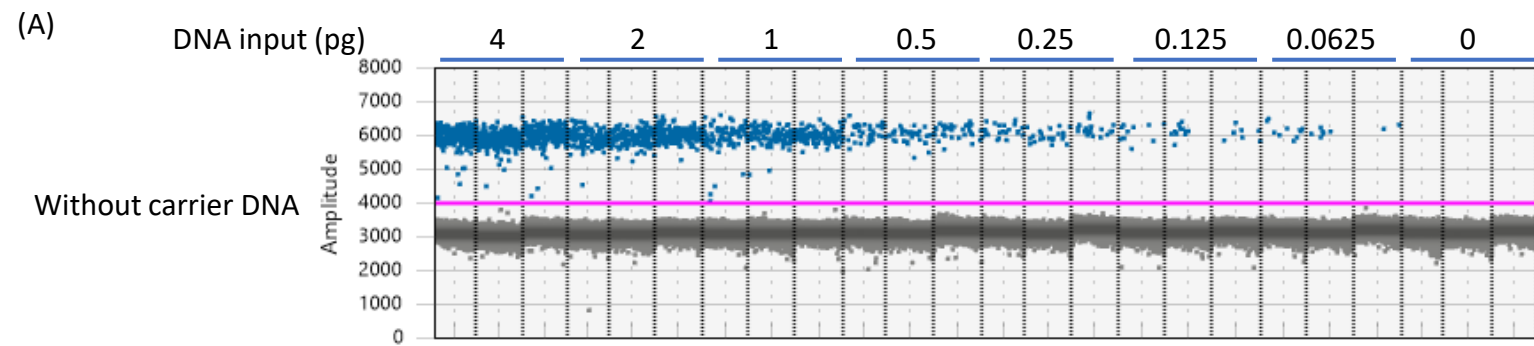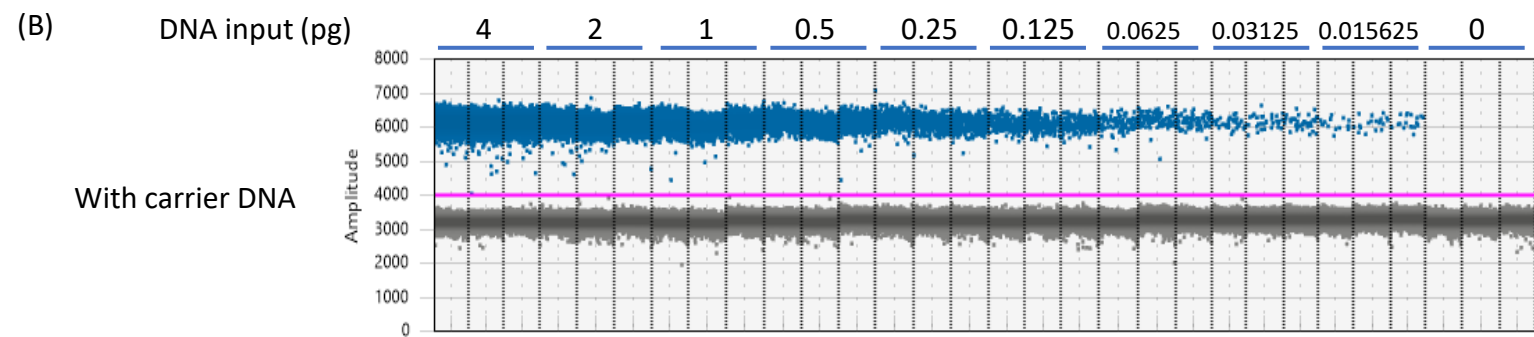

Supplement: S3 Fig — Series dilutions of C. albicans DNA were conducted in water or carrier DNA in parallel and assayed by duplex ddPCR in triplicate. The 1D amplitude plots were shown. (PDF) [file pone.0341560.s005.pdf]

**Multiplex  
Supermix  
w/ DTT**

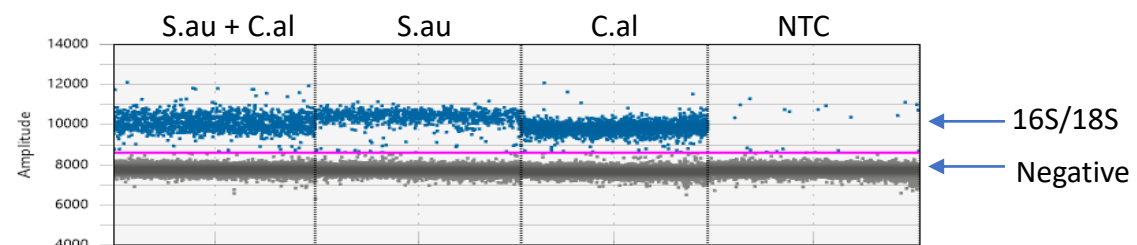

**Multiplex  
Supermix  
w/o DTT**

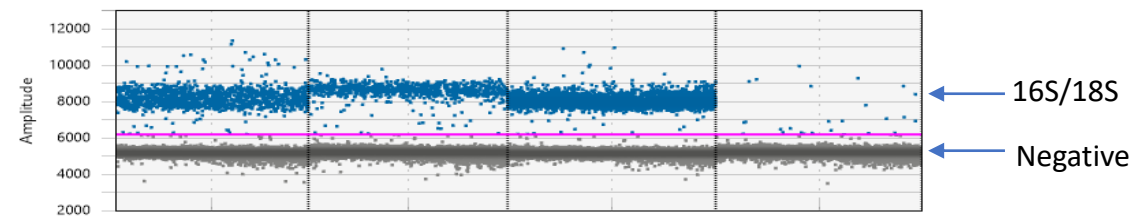

Supplement: S4 Fig — S.au, S. aureus. C.al, C. albicans. S.au and C.al gDNA was diluted in water and 4 pg of each sample was tested individually or combined, in the presence or absence of DTT. The 1D (left) and 2D (right) amplitude plots were shown. (PDF) [file pone.0341560.s006.pdf]
