# Supplementary material for: Development of a multiplex ddPCR assay for simultaneous absolute quantification of bacterial, fungal, and human DNA
Source: PLoS One. 2026 Feb 20;21(2):e0341560. doi: 10.1371/journal.pone.0341560 (PMC12923063; doi:10.1371/journal.pone.0341560)
Supplement: S2 Table — (PDF) [file pone.0341560.s004.pdf]

S2 Table. Detection of *S. aureus* and *C. albicans* genomic DNA by ddPCR in multiple independent runs

	***S. aureus* 16S**	***C. albicans* 18S**
Number of replicates	22	21
Average ddPCR concentration (copies/µL)	46.80	135.28
Coefficient of variation (CV%)	22.66	79.82
Minimum ddPCR concentration (copies/µL)	27.60	16.60
Maximum ddPCR concentration (copies/µL)	67.40	325.00

Genomic DNA from *S. aureus* and *C. albicans* was diluted in water and 4 pg of each sample was tested by the triplex or duplex ddPCR assay in multiple independent runs. The primer/probe concentrations of 16S were kept at 900/250 nM, and those of the 18S were kept at 450/125 nM.
